# Supplementary material for: Graph construction method impacts variation representation and analyses in a bovine super-pangenome
Source: Genome Biol. 2023 May 22;24:124. doi: 10.1186/s13059-023-02969-y (PMC10204317; doi:10.1186/s13059-023-02969-y)
Supplement: Supplementary file 1 — Additional file 1: Fig. S1. Pangenome repeat content. Fig. S2. Decomposing minigraph pangenomes. Fig. S3. Impact of adding P-lines to minigraph pangenomes. Fig. S4. Conflicting variation in three pangenomes. Fig. S5. Overlap of SVs between the three pangenomes and assemblies. Fig. S6. Variation representation accuracy in three pangenomes. Fig. S7. Pangenome performance on simulated data. Fig. S8. Tandem repeats that disagree between the three pangenomes. Fig. S9. VNTR representation in three pangenomes. Fig. S10. Representation of highly repetitive VNTR loci in three pangenomes. Fig. S11. Detection of two VNTR haplotypes in Original Braunvieh and Brown Swiss cattle. Fig. S12. eQTL mapping between the VNTR haplotype and normalized expression of 21,853 genes. Fig. S13. cis-expression QTL mapping for two genes and four lncRNA that were significantly associated with the VNTR haplotype. Fig. S14. Community detection from all-versus-all alignment of the 29 autosomes. [file 13059_2023_2969_MOESM1_ESM.pdf]

Fig. S1: **Pangenome repeat content.**

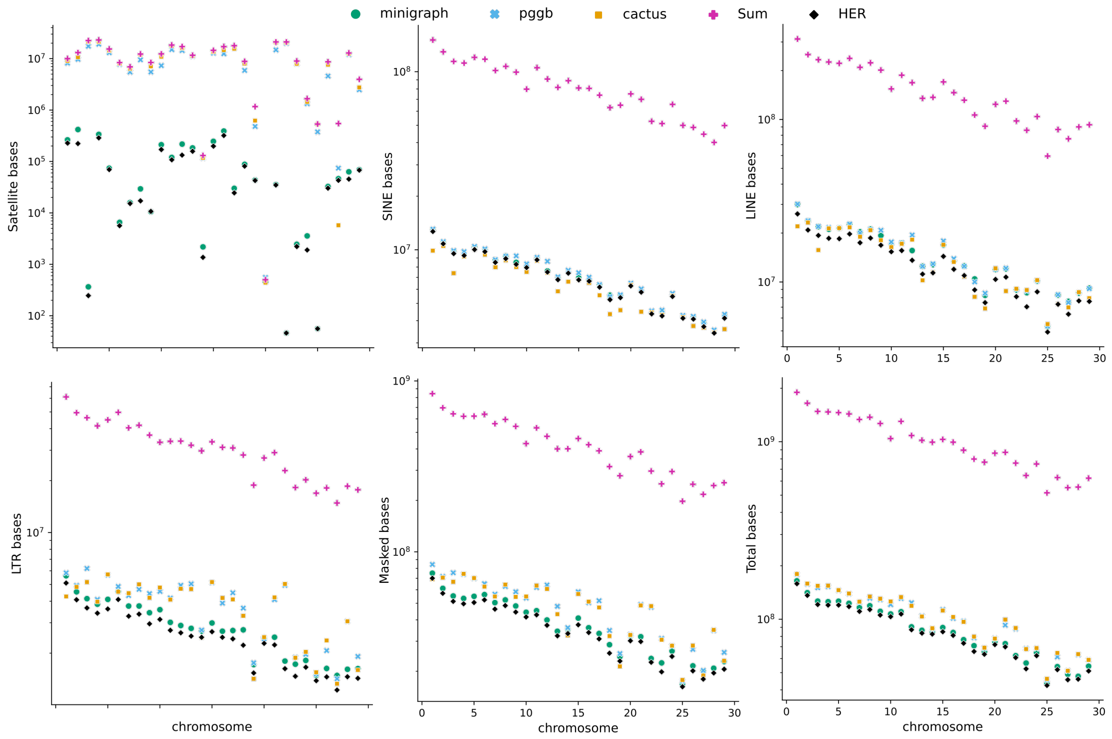

Repetitive sequence content of the three pangenomes, the sum across all 12 input assemblies (Sum), and the reference genome (HER). Many repetitive elements appear to be compactly represented by the pangenomes, and hence are substantially below the sum of the assemblies. However, centromeric sequences (Satellite) are either too biologically diverged or current mapping/smoothing algorithms are insufficient to collapse sequence from multiple assemblies into fewer nodes. As expected, minigraph contains effectively only centromeric sequence from the reference backbone and is unable to incorporate centromeric sequence that extends beyond the backbone.

Fig. S2: **Decomposing minigraph pangenomes.**

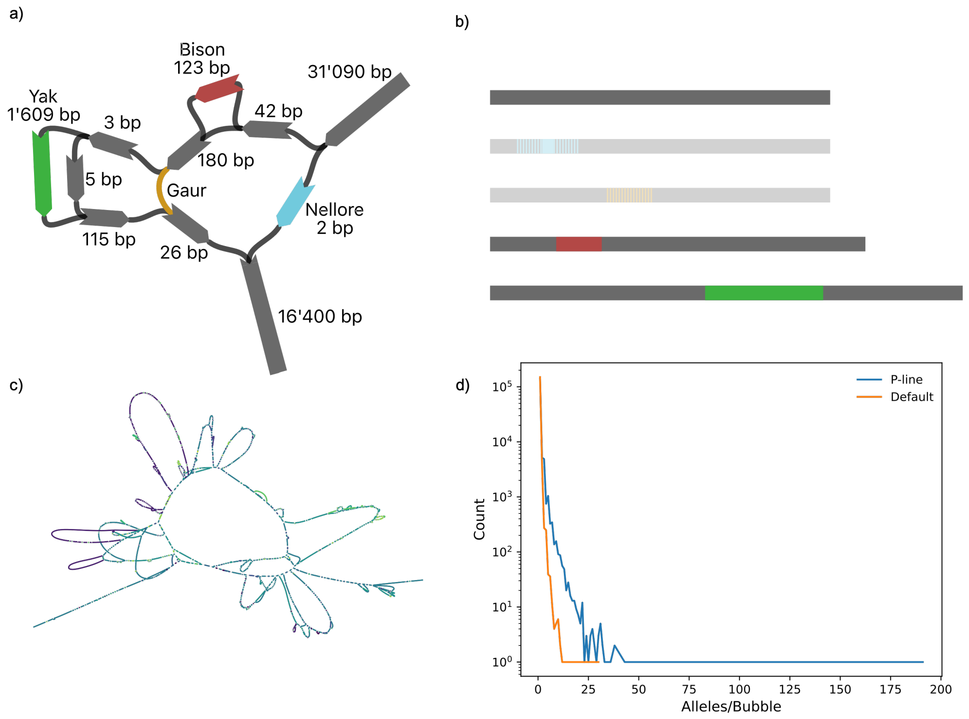

a) Complex bubble in minigraph (BTA29: 2250204-2250575) where reference nodes are dark gray and nonreference nodes (and nonreference deletions) are colored. b) Of the 5 alleles (including reference) observed in the graph, the faded Nellore and Gaur deletions are not recovered when deconstructing the minigraph pangenome without path information into VCF, leaving only 2 insertion and the reference alleles. When path information is added, all 5 alleles are correctly recovered. Minigraph by default does not include P-lines, which has a large detrimental effect on calling SVs in multi-allelic bubbles. c) A complex region (BTA27:6357301-7209140) produces 201 SV alleles without path information, and 462 SV alleles with P-lines. d) Including P-lines greatly increases the number of alleles found per bubble compared to default minigraph with no path information. The effect is magnified for complex bubbles containing many nodes and edges.

Fig. S3: **Impact of adding P-lines to minigraph pangenomes.**

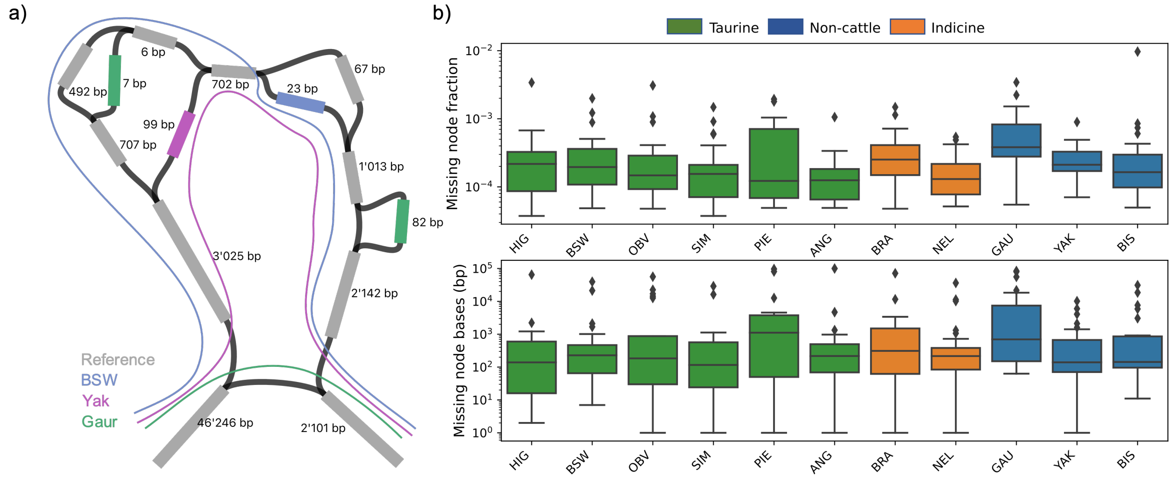

Adding P-lines post-hoc can lead to unexpected realignments. a) An extreme example bubble containing non-reference nodes for BSW, yak, and gaur. Realignment correctly traces BSW and yak through the appropriate non-reference nodes, while gaur is aligned through a deletion, incorrectly tracing the path which should include the two gaur non-reference nodes. As realignment produces an incorrect path, variant calling incorrectly assigns gaur a single 8,091 bp deletion as opposed to 485 bp deletion (top left) and 82 bp insertion (right). b) Some taurine, indicine, and non-cattle nodes included in minigraph pangenomes do not appear in realignment (top), although they do not account for a substantial portion of non-reference sequence found in the graphs (bottom).

Fig. S4: **Conflicting variation in three pangenomes**.

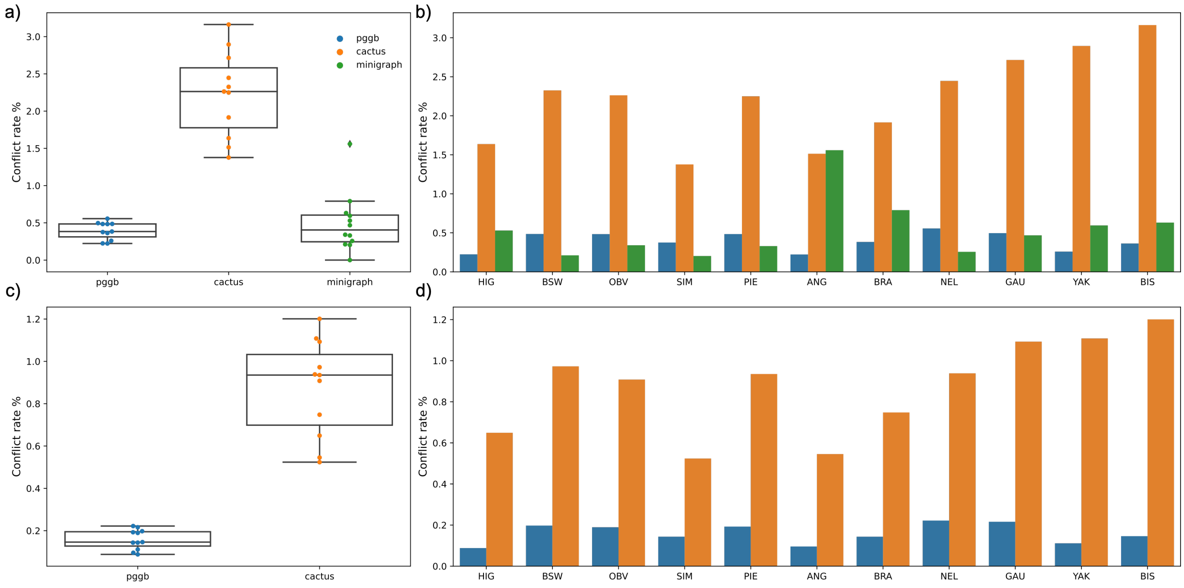

Proportion of conflicting genotypes detected from the three pangenomes from realigned assemblies for SVs (a) and small variations (c). Each point in the boxplots and every bar indicates the proportion of conflicting genotypes for each assembly. Conflict rates for cactus and pggb were determined by the CONFLICT tag assigned by vg deconstruct. Conflict rates for minigraph were determined during the post hoc realignment to add P-lines, when an assembly had a no call over a bubble. Per-sample conflict rates are shown for SVs (b) and small variation (d).

Fig. S5: **Overlap of SVs between the three pangenomes and assemblies.**

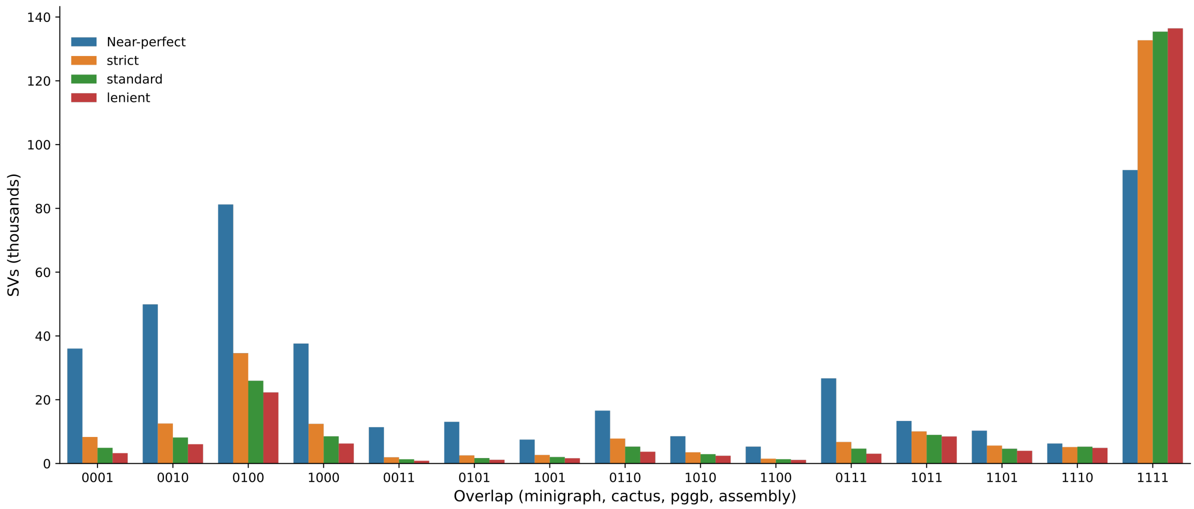

Near-perfect overlaps require exact basepair-level resolution and >85% sequence identity to merge SVs, while strict merges variants within 5 bp and >50% sequence identity. Almost all SVs that can be merged at the most lenient level are merged at this strict level, showing most SVs are highly similar between the pangenomes and assemblies.

Fig. S6: **Variation representation accuracy in three pangenomes.**

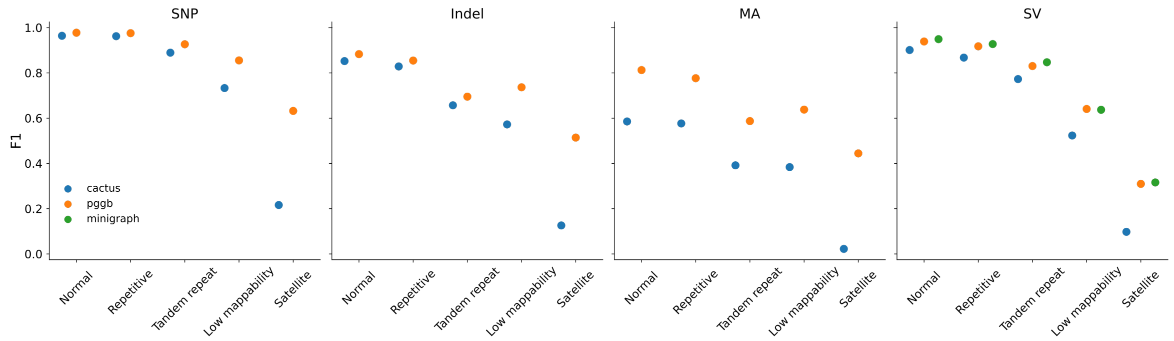

F-scores for SNPs, indels, multiallelics (MA), and SVs for minigraph, pggb, and cactus across the 5 region classifications using assembly-based variant calls as truth. The genomic region classification BED file is available online at 10.5281/zenodo.7737904.

Fig. S7: **Pangenome performance on simulated data.**

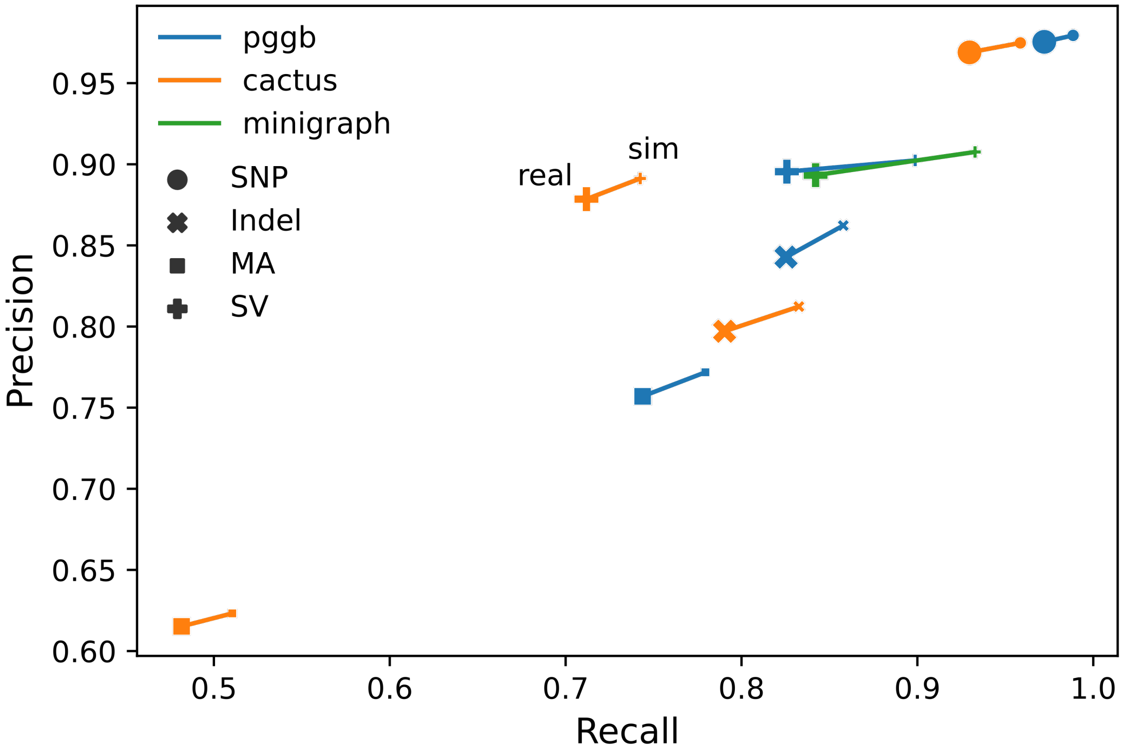

Real versus simulated (sim) assembly variant performance for pggb, cactus, and minigraph for SNPs, small indels (< 50bp), multiallelics (MA), and SVs (> 50bp). In all cases pangenomes built from simulated assemblies had slightly higher precision and recall.

Fig. S8: **Tandem repeats that disagree between the three pangenomes.**

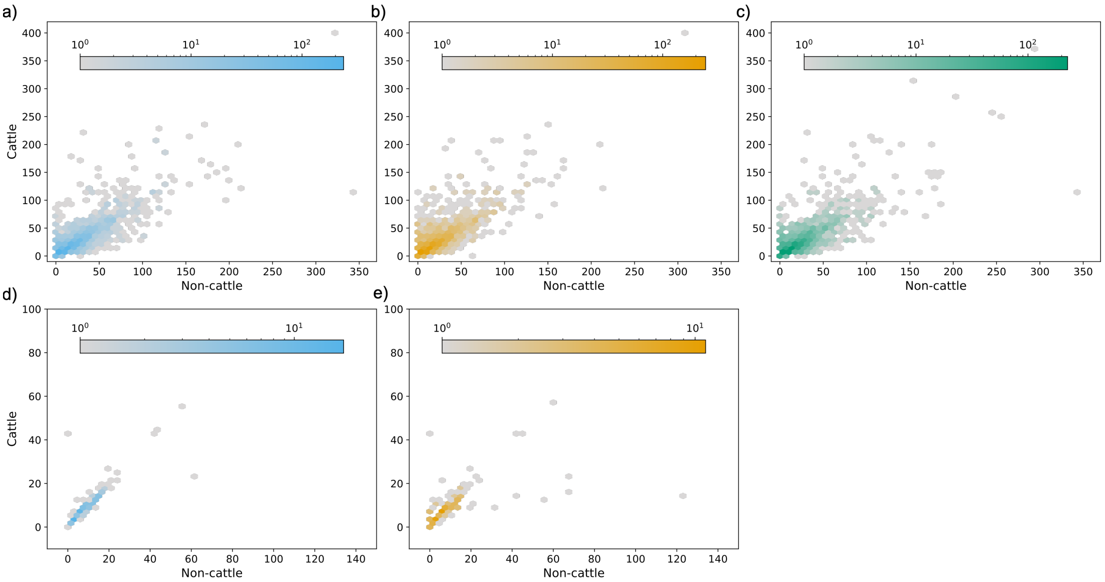

a), b), c) TR counts that did not agree across all the pangenomes are still broadly similar for pggb, cactus, and minigraph respectively. d), e) Similarly, TRs present in pggb and cactus but not minigraph that disagreed on count are still broadly similar and exhibit low variability between cattle and non-cattle.

Fig. S9: **VNTR representation in three pangenomes.**

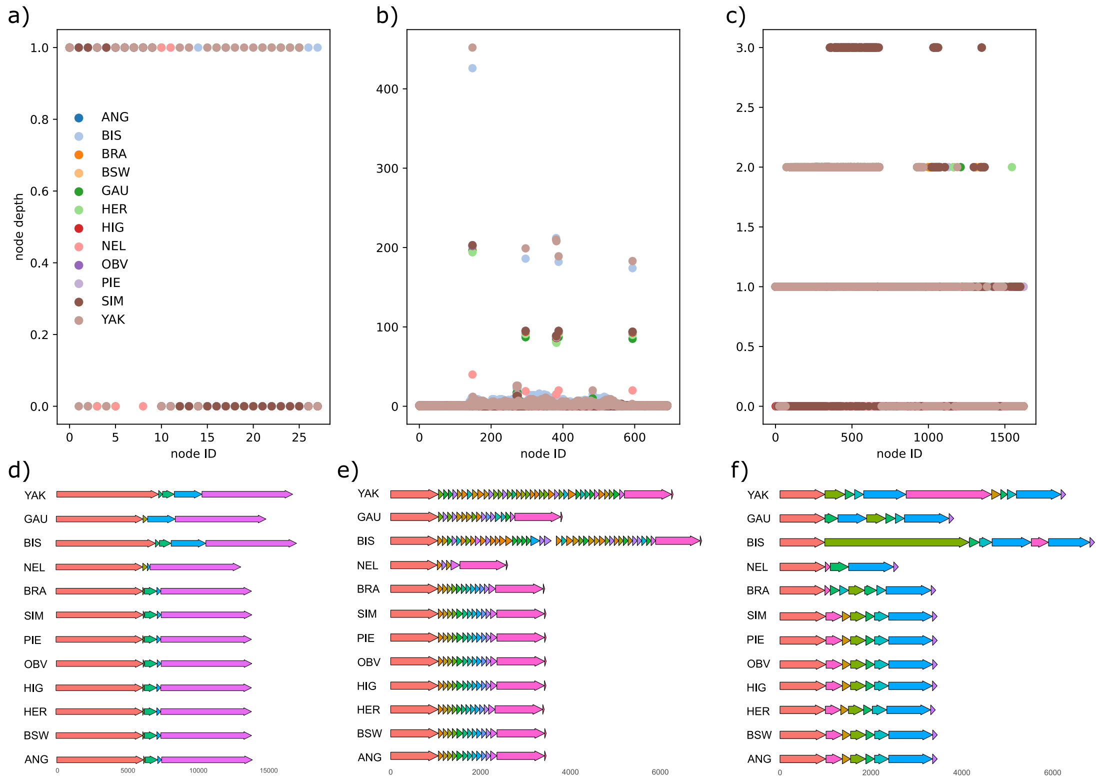

(a-c) Node depth per sample for minigraph, pggb, and cactus respectively over the VNTR described in Figure 6 including 1000 bp flanking sequence on both sides. Higher node depth implies samples looping through the same set of nodes, most apparent in pggb for Yak and Bison, who both had high counts of the tandem repeat. (d-f) Approximate linearised structure from the minigraph, pggb, and cactus pangenomes respectively for the subgraph from (a-c). No structures are reused within the same sample by minigraph (d), and only a few are reused by cactus (f). Pggb often reuses part of the graph structure, again most obvious for Yak and Bison.

Fig. S10: **Representation of highly repetitive VNTR loci in three pangenomes.**

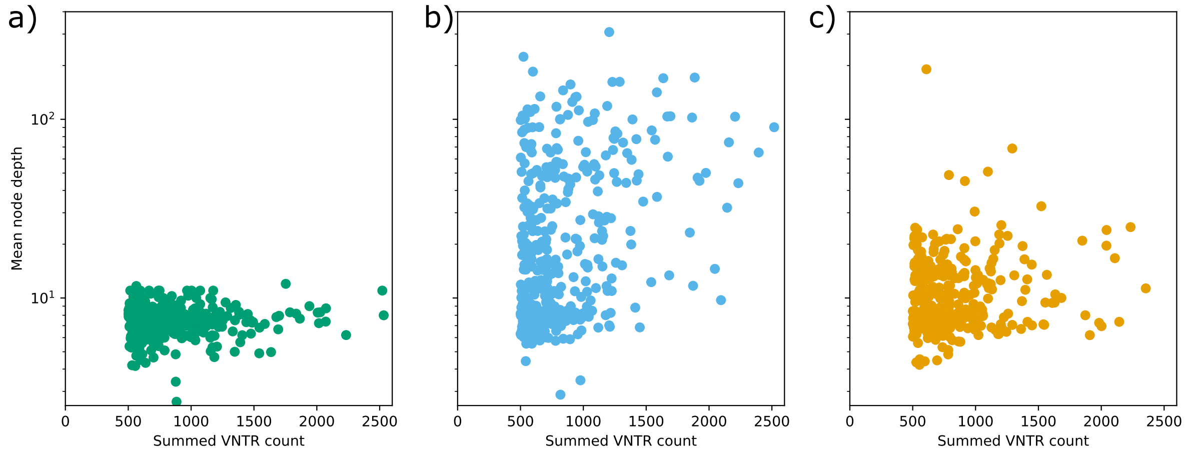

Mean node depth for minigraph (a), pggb (b), and cactus (c) at VNTR regions with high counts (summing counts over the 12 assemblies > 500). Minigraph never exceed a depth of 12 (1 use of the node per sample), while cactus and pggb regularly used the same node multiple times for the same sample.

Fig. S11: **Detection of two VNTR haplotypes in Original Braunvieh and Brown Swiss cattle.**

a)

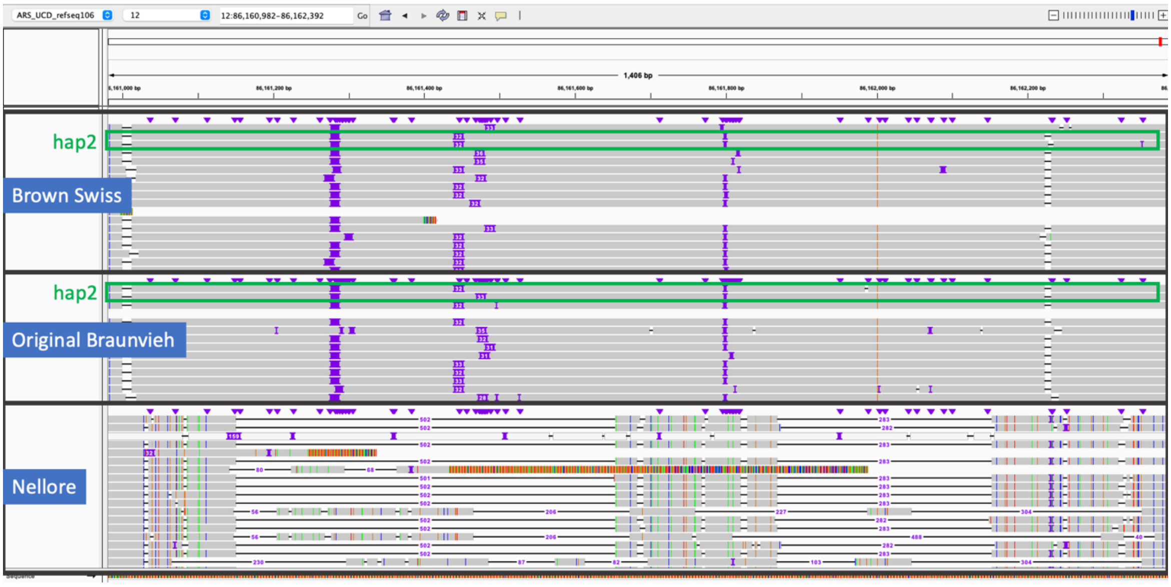

b)

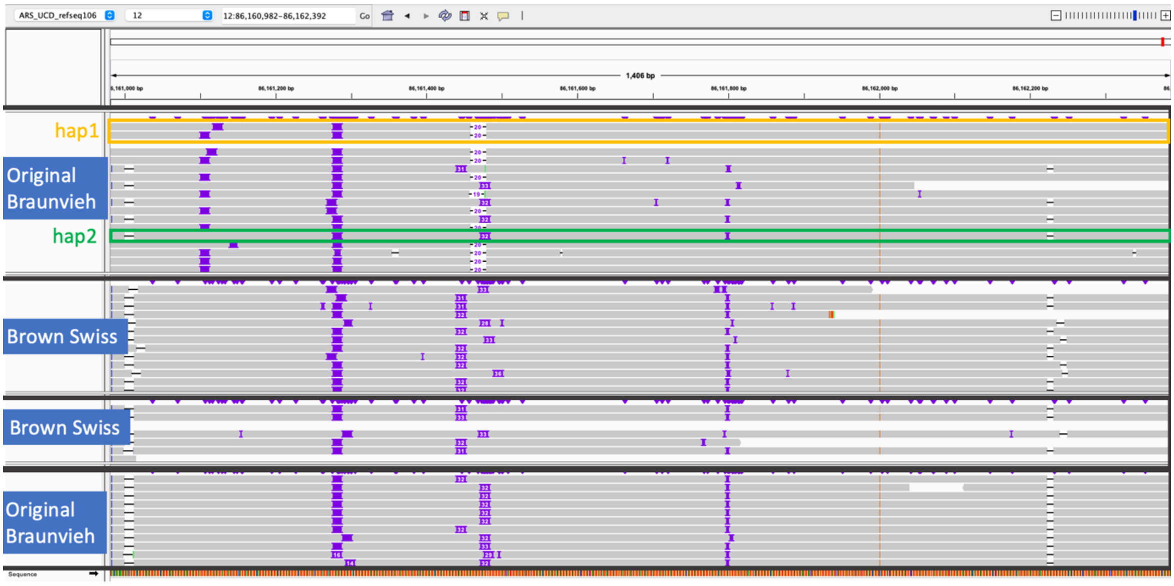

a) IGV screenshots of alignments from parental-binned HiFi reads of Brown Swiss, Original Braunvieh, and Nellore haplotypes that are included in the pangenomes. The screenshots cover the VNTR region (chr12:86,160,982-86,162,392). The Brown Swiss and Original Braunvieh haplotypes that are part of the pangenomes carry haplotype “hap2”. The Nellore haplotype is considerably shorted and diverged from the taurine haplotypes. b) We observe a second VNTR haplotype (“hap1”) in HiFi read alignments of four additional Original Braunvieh and Brown Swiss animals.

Fig. S12: **eQTL mapping between the VNTR haplotype and normalized expression of 21,853 genes.**

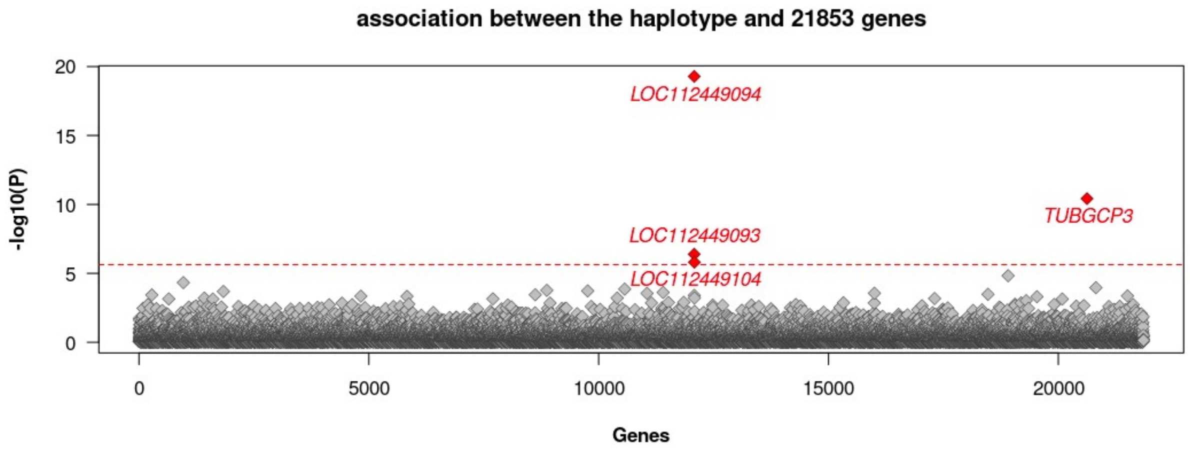

Genes and ncRNA are ordered in alphabetical order. The red line indicates the Bonferroni-corrected significance threshold (P=0.05 / 21853). Genes and ncRNA exceeding the Bonferroni-corrected threshold are coloured on red.

Fig. S13: cis-expression QTL mapping for two genes and four lncRNA that were significantly associated with the VNTR haplotype.

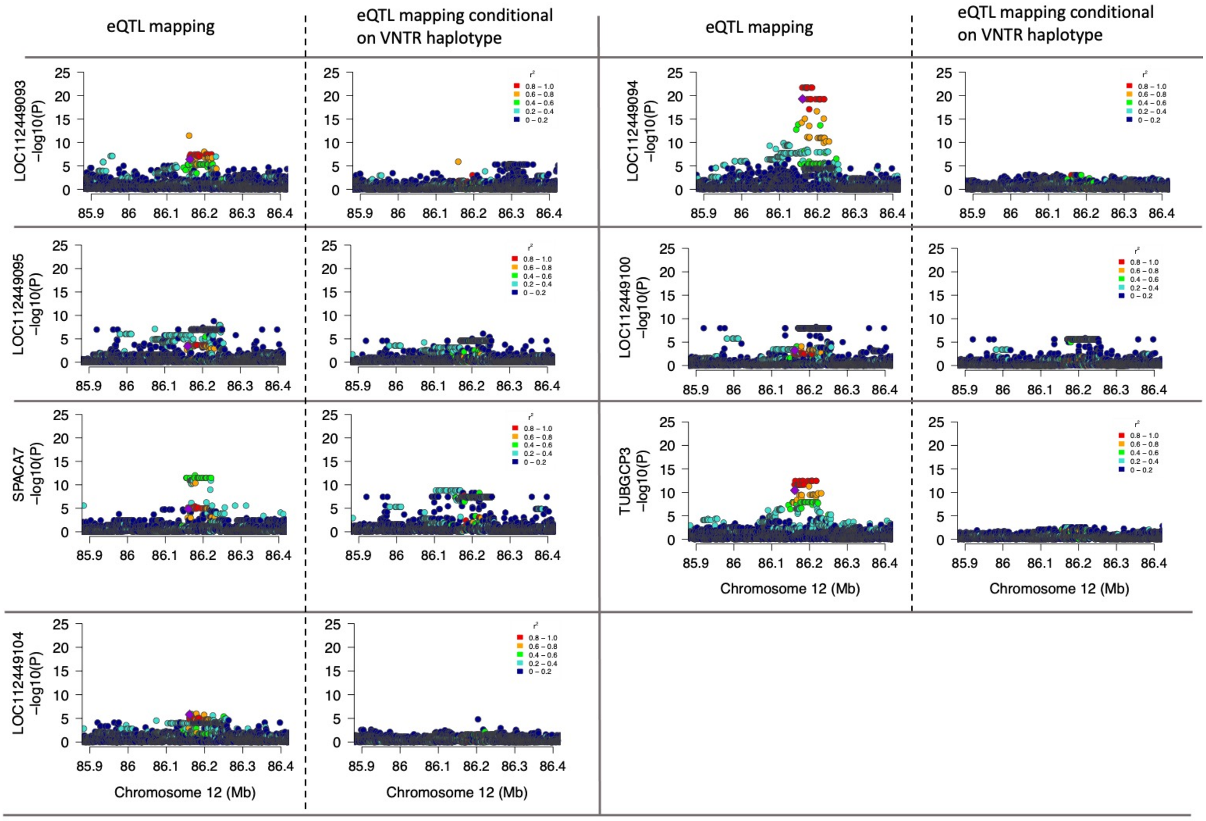

Variants within 1Mb of the genes were tested for association. Different colours indicate the pairwise linkage disequilibrium (r^2) between the VNTR haplotype (violet) and all other variants. The cis-eQTL mapping was repeated conditional on the VNTR haplotype.

Fig. S14: **Community detection from all-versus-all alignment of the 29 autosomes.**

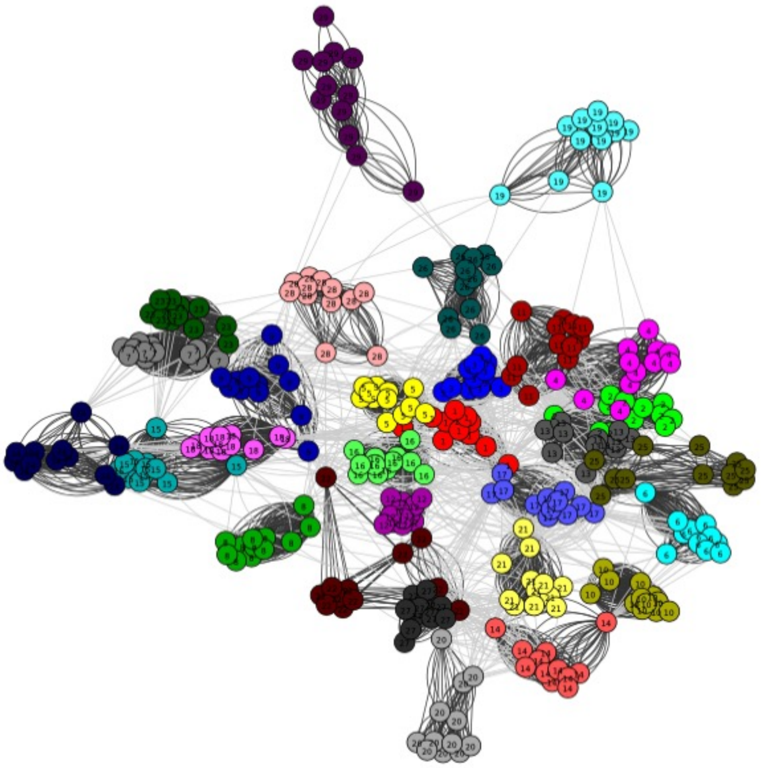

Although there are links between different chromosomes (implying inter-chromosomal homology), all detected communities consisted of only single autosomes, implying there were no strong inter-chromosomal events present.
