# Supplementary material for: Graph construction method impacts variation representation and analyses in a bovine super-pangenome
Source: Genome Biol. 2023 May 22;24:124. doi: 10.1186/s13059-023-02969-y (PMC10204317; doi:10.1186/s13059-023-02969-y)
Supplement: Supplementary file 2 — Additional file 2: Table S1. Compute resources for pangenome deconstruction. Cumulative CPU hours and maximum memory needed for vg deconstruct and the number of variants recovered for the three pangenomes. [file 13059_2023_2969_MOESM2_ESM.pdf]

Tool	CPU hours	Memory (GB)	Variants
minigraph	0.2	0.5	164,723
pggb	10.1	11.1	57,350,905
cactus	28.3	12.8	61,028,922
