# Supplementary material for: Graph construction method impacts variation representation and analyses in a bovine super-pangenome
Source: Genome Biol. 2023 May 22;24:124. doi: 10.1186/s13059-023-02969-y (PMC10204317; doi:10.1186/s13059-023-02969-y)
Supplement: Supplementary file 3 — Additional file 3: Table S2. Assessment of structural variant representation with orthogonal data. Orthogonal optical mapping (OM) and ONT data from relevant breeds/species to assess SV overlap with three pangenomes. Optical mapping generates fewer SV calls, and so only the total number of overlapping SVs are shown. ONT reads generated a comparable number of SVs to the pangenomes, and we can directly calculate an F-score. The Nellore samples are unrelated to the NEL haplotype used in this work, while the remaining data are from the same individual used to generate the assembly. [file 13059_2023_2969_MOESM3_ESM.pdf]

| Sample | Technology | Coverage | Minigraph | Pggb | Cactus | Assembly |
| --- | --- | --- | --- | --- | --- | --- |
| Nellore | OM | 200x | 813 | 780 | 597 | 744 |
| ANG | OM | 80x | 191 | 177 | 153 | 158 |
| BRA | OM | 80x | 351 | 316 | 245 | 295 |
| OBV | ONT | 17x | 0.763 | 0.773 | 0.709 | 0.803 |
| BSW | ONT | 55x | 0.777 | 0.780 | 0.713 | 0.812 |
| PIE | ONT | 83x | 0.746 | 0.757 | 0.693 | 0.780 |
| NEL | ONT | 48x | 0.840 | 0.842 | 0.786 | 0.893 |
| GAU | ONT | 73x | 0.871 | 0.860 | 0.816 | 0.918 |
