# Supplementary material for: Graph construction method impacts variation representation and analyses in a bovine super-pangenome
Source: Genome Biol. 2023 May 22;24:124. doi: 10.1186/s13059-023-02969-y (PMC10204317; doi:10.1186/s13059-023-02969-y)
Supplement: Supplementary file 5 — Additional file 5: Table S4. Impact of parameter settings on graph alignment. GraphAligner alignments using relaxed (-x dbg -C 100000 --max-trace-count 5 --seeds-minimizer-ignore-frequent 0.001 --precise-clipping 0.9) or strict (-x vg) alignment parameters. The edit rate and query coverage are taken from a single 500 Kb window in chromosome 12 of the Brown Swiss assembly (start at 60867381), chosen for its complexity and subsequent poor relaxed alignment. CPU time is given in seconds and Memory is peak RAM usage in GB. Strict alignment quickly requires more resources for pggb and becomes prohibitive for cactus for whole chromosome alignment. [file 13059_2023_2969_MOESM5_ESM.pdf]

	Relaxed				Strict			
	Edits	Query coverage	CPU	Memory	Edits	Query coverage	CPU	Memory
minigraph	2285	99.6	50	0.7	2285	99.6	99	1.0
cactus	0	0	164	6.9	18	100	244	14.1
pggb	0	0	140	5.1	17	100	189	8.1
