# Supplementary material for: Graph construction method impacts variation representation and analyses in a bovine super-pangenome
Source: Genome Biol. 2023 May 22;24:124. doi: 10.1186/s13059-023-02969-y (PMC10204317; doi:10.1186/s13059-023-02969-y)
Supplement: Supplementary file 6 — Additional file 6: Table S5. Compute resources for alignment of additional assemblies. Compute resources for aligning the 12 assemblies included in pangenome construction and the 8 held out for analysis. CPU hours are averaged per assembly for both the bulk and centro-/telomeric regions, and memory is the peak RAM usage across all assemblies. [file 13059_2023_2969_MOESM6_ESM.pdf]

	CPU hours				Memory (GB)			
	Included		Held-out		Included		Held-out	
	Bulk	Centro-/telo	Bulk	Centro-/telo	Bulk	Centro-/telo	Bulk	Centro-/tel
pggb	3.7	2.4	121.1	1.8	31.7	109.8	108.2	79.6
cactus	5.6	0.9	73.2	1.3	130.4	99.4	349.5	126.9
minigraph	2.6	1.3	2.8	1.3	4.5	4.3	20.8	1.9
