# Supplementary material for: Graph construction method impacts variation representation and analyses in a bovine super-pangenome
Source: Genome Biol. 2023 May 22;24:124. doi: 10.1186/s13059-023-02969-y (PMC10204317; doi:10.1186/s13059-023-02969-y)
Supplement: Supplementary file 8 — Additional file 8: Table S7. Several obvious misalignments found in different pangenomes which affects TR count genotyping. Part of the size difference may be true if there is variation in the number of tandem repeats, but the obvious majority of the large mis-translated regions are due to repetitive regions or spurious graph cycles. Δ bp is the size difference for the lifted-over sample TR coordinates from the original reference TR coordinates. [file 13059_2023_2969_MOESM8_ESM.pdf]

Pangenome	Sample	Chromosome	Reference coordinates	Sample coordinates	Δ bp
minigraph	YAK	5	118527963-118528562	118396757-118426843	29.5 Kb
pggb	NEL	13	11057154-11057352	16184369-23015882	6.8 Mb
cactus	NEL	1	121617962-121618026	58878820-125260767	66.4 Mb
cactus	SIM	4	3319408-3319495	3310542-113157354	109.8 Mb
cactus	BIS	8	71781915-71781975	23187990-108691932	85.5 Mb
cactus	BIS	12	6381693-6381754	657155-36976359	36.3 Mb
cactus	GAU	12	6381693-6381754	439517-36663196	36.2 Mb
cactus	YAK	12	6381693-6381754	107663-36390629	36.3 Mb
