# Supplementary material for: Graph construction method impacts variation representation and analyses in a bovine super-pangenome
Source: Genome Biol. 2023 May 22;24:124. doi: 10.1186/s13059-023-02969-y (PMC10204317; doi:10.1186/s13059-023-02969-y)
Supplement: Supplementary file 9 — Additional file 9: Table S8. Minigraph pangenomes constructed with different “minimum variant length” parameter (L) values. CPU hours required for pangenome construction increased dramatically for L smaller than 10 bp. Warnings refer to the number of “impossible insert” warnings issued during pangenome construction, relating to unsuitable graph topology. Bubbles and Nodes respectively refer to the number of top-level bubbles and nodes present across the autosomes. VNTR overlaps is the number of VNTRs (in total 9,568) that overlap with a graph bubble. [file 13059_2023_2969_MOESM9_ESM.pdf]

L (bp)	CPU hours	Warnings	Bubbles	Nodes	Non-reference bases (Mb)	VNTR overlaps
50	13.45	131	153,865	425,245	108.85	5,742
30	14.80	152	220,761	611,795	110.66	6,552
10	15.32	222	709,032	1,977,837	118.07	7,613
5	29.35	484	1,571,642	4,613,837	128.20	7,898
2	53.37	24,678	8,125,250	26,623,047	220.19	8,441
