# Supplementary material for: Graph construction method impacts variation representation and analyses in a bovine super-pangenome
Source: Genome Biol. 2023 May 22;24:124. doi: 10.1186/s13059-023-02969-y (PMC10204317; doi:10.1186/s13059-023-02969-y)
Supplement: Supplementary file 10 — Additional file 10. Review History. [file 13059_2023_2969_MOESM10_ESM.docx]

Review History

**First round of review**

**Reviewer 1**

**Were you able to assess all statistics in the manuscript, including the appropriateness of statistical tests used?** Yes, and I have assessed the statistics in my report.

**Comments to author:**

The manuscript by Leonard et al. evaluated three popular graph construction methods and their impacts on structural variation representation and analyses using cattle and related genomes. I feel that its results and methods are not fully validated, and its ground truths are somehow weak. I have three major comments:

1. All reported raw data, including the three resultant pangenomes, orthogonal optical mapping data, as well as WGS and RNA-seq data for eVNTR mapping, must be deposited in well-known depositories like EBI ENA.

2. Based on the below, if no human analysis is feasible, at least a validation using cattle SV simulations needs to be performed to compare three pangenomes.

3. Since there are no cattle equivalents like results generated y the human Genome in a Bottle Consortium (GIAB) or the Human Genome Structural Variation Consortium (HGSVC), there is no ground truth to gauge the results. Are there any similar benchmarking studies performed in humans? If yes, please cite them and compare this study to see what is shared and what are the novel findings. If not, including CHM13, HG0002, and other dozens of human genomes in trios may be a better place to first perform this benchmarking study.

Other comments:

4. More texts about human literature should be included in the Introduction. Plants can be introduced in the Introduction also.

5. For example, the validation with optical mapping is not enough: "Pggb had the largest number of validated SVs (1,661, 0.97%), followed closely by minigraph (1,656, 0.98%) and assembly (1,584, 0.97%) whereas cactus (1,218, 0.66%) had lower support." However, all of the validation percentages were <1%, which was not satisfactory.

6. The assembly-based SV calls are not fully validated either. "All three pangenomes contained more SVs than the assembly-based "truth", with many private SVs in minigraph (8.5k), pggb (8.1k), and cactus (25.9k), likely representing false positives, agreeing with recent findings in human pangenomes [13]."

7. Dividing genomes into subregions is too simplistic: repetitive vs. non-repetitive and these "centro-/telomeric" regions separately to the rest of the "bulk" sequence. The human genome is better classified into over ten types of different genomic regions as defined by GIAB: such as GC content, mappability, Low Complexity, Segmental Duplications, Functional Technically Difficult, Genome Specific, Functional Regions, Other Difficult, Union, Ancestry, and XY. Some of them, including GC content, mappability, Low Complexity, and others, are available for the cattle genomes and can be fine-mapped and better assessed.

8. Some sentences are kinds of speculations, lacking quantitative measurements. "However, there was a weak increase in edit rate in pggb and cactus pangenomes for HiFi assemblies, potentially related to the increased resolution of complex and repetitive areas beyond centro-/telomeric sequence that cannot be realigned accurately." Please see also "We found that approximately 0.5% and 3.9% of alleles in pggb and cactus were removed during preliminary filtering when only keeping alleles seen in genotypes, suggesting variants may either exist in complex regions that cannot be genotyped confidently or strongly tangled regions that produce spurious variant calls."

**Reviewer 2**

**Were you able to assess all statistics in the manuscript, including the appropriateness of statistical tests used?** Yes, and I have assessed the statistics in my report.

**Comments to author:**

This manuscript describes the attempt to create a super-pangenome with bovine species with three pangenome graph construction approaches, pggb, minigraph and cactus. The goal of the manuscript is to evaluate the accuracy of representation of the pangenomes by each approach and make suggestions on different application scenarios.

This manuscript evaluates the three approaches by

1. The computing resources (time and memory) required to construct the data structures

2. The consensus of representation of SVs and SNPs with the assembly-based "truth"

3. General accuracy of base-level representation in edit rate from realigning input assemblies

4. The capabilities of representing polymorphic VNTR loci.

Overall, the manuscript is clear and concise on the metric and objective in each comparison. However, the conclusion drawn from the comparisons are not very convincing for the following reasons: (1) for SVs, since there is no ground truth or a validated set of SVs, it is difficult to tell which set of SVs are true SVs. (2) A lot of alternative approaches to build pangenomes are overlooked. For example, colored de Bruijn graphs and RLZ-graphs are structures that can also take complete assemblies as input. The output of both genome graph construction schemes can be used with GraphAligner. On the other hand, pangenomes can be represented by not only genome graphs but as a set of strings (see reference flow, Moni and prefix-free parsing works for examples). Without benchmarking with a more diverse set of methods to construct the pangenome, the result of this manuscript is not complete. (3) Given a set of assemblies, there are multiple ways of selecting the samples to be included or held out for analysis. This manuscript only selected one way and does not give a justification for the choice. It would be equally interesting and important to test whether the diversity and the number of the species to be included in the pangenome affect the comparison metrics.

Some minor comments:

- It would be very helpful to determine the quality of the comparison results if centromeric and telomeric regions are excluded.

- In the section "consensus of genomic variation". Are the additionally recovered SVs from the minigraph related to the realignments that are incongruent with graph topology? Seems that minigraph is creating or removing sequences arbitrarily that may result in the additional SVs.

- How does the merging approach influence the overlap of SVs? Is it the different parameters or something else? Please clarify.

- Please add legend to Figure 2(c) or reorder the figures.

- When they compare optical mapping with the pangenomes, the overlap is low because the OGM is from unrelated samples. Is it possible to calculate the overlap based on the covered breed only? Instead of providing numbers <1%, it would be helpful to see, for the covered breeds, what are the support percentage.

- Another important question that comes with using a pangenome is whether using a pangenome improves the short-read/long-read/any-read alignment ratio. It would be good that this work also evaluate this by comparing to aligning to the default linear reference genome.

- For VNTRs, it would be helpful to give an overview of how each graph stores repeats. Do they identify the repeat motif and create self-loops, or do they store the entire VNTR region as is?

- If pangenomes were constructed per-chromosome, would there be missed inter-chromosomal structural variants?

Please consider the following questions:

- Are the methods appropriate to the aims of the study, are they well described, and are necessary controls included? If not, please specify what is required.

The use of a pangenome graph is not essential to many of the analysis (e.g. finding SVs, VNTRs and analyzing regulatory impacts of VNTRs). Especially for VNTR, since the genome graphs are constructed by assemblies, there is no need to conduct a genotyping step as there are ample prior knowledge about the origin of the VNTRs. It is also not necessary to go through the time-consuming steps to construct and align the assemblies in the first place. The entire section about VNTR analyses seems irrelevant to the original challenges identified in the manuscript.

- Are sufficient details provided to allow replication and comparison with related analyses that may have been performed? If not, please specify what is required.

Yes.

- Does the work represent a significant advance over previously published studies?

Yes. It is interesting to see that pangenome approaches are applied to non-human species and to more diverse species that are more different.

- Is the paper of broad interest to others in the field, or of outstanding interest to a broad audience of biologists?

Yes. It provides some insights into the application of pangenome graphs and some analyses that could be done on it aside from just read alignment.

**Authors Response**

**Point-by-point responses to the reviewers’ comments:**

Reviewer #1: The manuscript by Leonard et al. evaluated three popular graph construction methods and their impacts on structural variation representation and analyses using cattle and related genomes. I feel that its results and methods are not fully validated, and its ground truths are somehow weak. I have three major comments:

**1. All reported raw data, including the three resultant pangenomes, orthogonal optical mapping data, as well as WGS and RNA-seq data for eVNTR mapping, must be deposited in well-known depositories like EBI ENA.**

We agree with the reviewer on the importance of data availability and confirm that all raw data have been deposited in appropriate repositories. We have clarified our existing data availability statement to more clearly state that all raw data used in this manuscript are publicly available. Some data, e.g., the optical mapping data, were made public in other publications like Talenti et al. 2022 and have been cited to indicate so. The origins of the assemblies were cited in Table 3, but we have now made this more explicit in the data availability statement. It was not our intention to publish the pangenomes as the raw data, assemblies and our pipelines are all publicly available, but we have uploaded the pangenomes and genomic regions classification BED file to Zenodo for completeness at https://doi.org/10.5281/zenodo.7737904.

**2. Based on the below, if no human analysis is feasible, at least a validation using cattle SV simulations needs to be performed to compare three pangenomes.**

We address this point below.

**3. Since there are no cattle equivalents like results generated y the human Genome in a Bottle Consortium (GIAB) or the Human Genome Structural Variation Consortium (HGSVC), there is no ground truth to gauge the results. Are there any similar benchmarking studies performed in humans? If yes, please cite them and compare this study to see what is shared and what are the novel findings. If not, including CHM13, HG0002, and other dozens of human genomes in trios may be a better place to first perform this benchmarking study.**

We appreciate the reviewer’s comments, and we have expanded the literature references to similar work performed in human and plants that corroborate our results (addressed further in next comment). However, one specific aspect of our study was to examine these effects in a collection of assemblies with greater genomic diversity than human samples, as can be common in crop & livestock agriculture, conservation genomics, etc., and so we cannot simply use CHM13, HG002/3/4, and other human assemblies despite their more developed resources and availability of truth sets.

We have reworked the optical mapping analysis to better indicate the fraction of overlapping SVs relative to each breed (suggested by reviewer 2), as well as including additional optical mapping data available from the Brahman and Angus haplotypes that are part of the pangenomes. We also included additional orthogonal validation, using ONT long reads from 5 samples (Original Braunvieh, Brown Swiss, Piedmontese, Nellore, and gaur) to call SVs and compare the overlap with the pangenomes. While there are limited samples available, this process of using multiple independent technologies to assess SVs is comparable to the GIAB procedure, and so we are confident our assessment of pangenome SV accuracy is thoroughly supported. The reworked and new text is on Lines 183-191, and Supplementary Table 2.

*We used optical mapping from two Nellore samples [28] unrelated to the NEL haplotype and from the BRA and ANG haplotypes [29], as well as ONT long reads from the OBV, BSW, PIE, NEL, and GAU haplotypes [30] to further validate SV consensus in the pangenomes. Since optical maps call larger SVs than sequencing reads (typically minimum SV size of 1 Kb), and the ONT datasets had different read lengths, quality, and coverage, the overlap with the pangenome SVs was not expected to be complete. Both sets of orthogonal data substantiate that minigraph and pggb best represent SVs with nearly equal support (1355 and 1283 pangenome SVs overlapping optical map SVs, and ONT SV F-score of 0.799 and 0.802 respectively), while cactus had comparatively lower support (1045 optical map SV overlaps and ONT F-score of 0.743) (Supplementary Table 2).*

We also performed both SV and small variant simulation through pseudo-assemblies created from the ARS-UCD1.2 reference genome modified with breed/species-specific variation. We chose this approach as we are not aware of a standard method for simulating entire assemblies, and simulating SVs or small variation at the read level would require assembling the genomes which can introduce its own errors and would not be useful as a “higher truth set” than the original assemblies. The performance of pangenomes using simulated assemblies is only slightly higher than using the real assemblies, suggesting the original analysis is valid and the results are general. The new text is on Lines 211-219 including reference to a newly compiled Supplementary Figure 7.

*We also simulated assemblies for chromosome 29 by introducing known variation per sample to the ARS-UCD1.2 reference genome, removing uncertainty arising from variation that was not be called from the real assemblies when aligned to ARS-UCD1.2. Pangenomes constructed from these simulated assemblies had minor improvements to precision and recall for minigraph, pggb, and cactus, as well as for all types of variants (mean F1 improvement for SNP: 0.014, Indel: 0.027, multi-allelic: 0.023, SV: 0.039,* ***Supplementary Figure 7****) compared to pangenomes constructed from the real assemblies. The slight improvement suggests that hard-to-call regions or centromeric sequence (not included in simulated assemblies) hurt pangenome accuracy, but that there is still some loss of accuracy in pangenome construction or downstream conversion to VCF even with simulated assemblies.*

**Other comments:**

**4. More texts about human literature should be included in the Introduction. Plants can be introduced in the Introduction also.**

We have added additional literature for related studies conducted in multiple species like humans and plants. We also included some additional human literature discussing applications and uses of pangenomes with other datasets like short reads.

Lines 51-54:

*These pangenomes have revealed homology in acrocentric chromosomes (Guarracino et al., 2023), trait variation resulting from variable number tandem repeats (Lu et al., 2021), and complex structural variations (Porubsky et al., 2022). Pangenomes can leverage existing datasets like short reads to accurately genotype structural variants (Ebler et al., 2022).*

Lines 56-58:

*Pangenomes have also been adopted in many non-human species, revealing new clues to missing heritability in tomato (Zhou et al., 2022), and evolutionary and adaptation insights into potato (Tang et al., 2022) and sheep (Li et al., 2022), amongst others.*

**5. For example, the validation with optical mapping is not enough: "Pggb had the largest number of validated SVs (1,661, 0.97%), followed closely by minigraph (1,656, 0.98%) and assembly (1,584, 0.97%) whereas cactus (1,218, 0.66%) had lower support." However, all of the validation percentages were <1%, which was not satisfactory.**

We have reworked this section to calculate the overlaps relative to the breed-covered SVs. However, this overlap for Nellore is still between unrelated individuals and two orthogonal technologies (optical maps generally call SVs >1 Kb, where sequencing-based calls can be > 50 bp), and so the lower overlap percentage was a consequence of the direct comparison. We have extended this section with additional optical mapping, and now report only the total number of overlapping SVs to not confuse the reader with the low percentages. The additional validation with ONT reads a comparable number of SVs to the pangenomes, and we can use a comprehensive metric like F-score directly. The reworked and new text is on Lines 183-191, and Supplementary Table 2.

**6. The assembly-based SV calls are not fully validated either. "All three pangenomes contained more SVs than the assembly-based "truth", with many private SVs in minigraph (8.5k), pggb (8.1k), and cactus (25.9k), likely representing false positives, agreeing with recent findings in human pangenomes [13]."**

The assembly-based SV calls are determined using widely used approaches (minimap2 & paftools.js from Li 2018) and on high quality genomes, and so these SV calls are as near to optimal as possible with current algorithms. We have rewritten this discussion after more detailed reading of (Liao et al. 2022), now stating on Lines 430-432:

*All three pangenomes contained more SVs than the assembly-based “truth”, with private SVs in minigraph (8.5k), pggb (8.1k), and cactus (25.9k). Recent work in human pangenomes suggest many of these putative errors are SVs missed by linear reference-based calling [13], although the substantially larger number in cactus likely indicates many true errors.*

**7. Dividing genomes into subregions is too simplistic: repetitive vs. non-repetitive and these "centro-/telomeric" regions separately to the rest of the "bulk" sequence. The human genome is better classified into over ten types of different genomic regions as defined by GIAB: such as GC content, mappability, Low Complexity, Segmental Duplications, Functional Technically Difficult, Genome Specific, Functional Regions, Other Difficult, Union, Ancestry, and XY. Some of them, including GC content, mappability, Low Complexity, and others, are available for the cattle genomes and can be fine-mapped and better assessed.**

We thank the reviewer for this extremely helpful suggestion. We were unable to find such an annotation for region type in the ARS-UCD1.2 reference genome, and constructing a comprehensive list is a paper in its own right (see [<https://www.frontiersin.org/articles/10.3389/fgene.2015.00338/full>](https://www.frontiersin.org/articles/10.3389/fgene.2015.00338/full)) and beyond the scope of our current work. However, we created 5 classifications (centromeric satellites, tandem repeats, repetitive elements, low mappability, and “normal”), and these categories demonstrated significantly more variation in SV and small variation accuracy than our previous analysis. We have updated Figures 2 and 3 accordingly and rewritten the results section on line 148-154 with:

*We classified the ARS-UCD1.2 reference genome into 5 regions: centromeric satellites (0.13%), tandem repeats (6.21%), repetitive elements (35.78%), low mappability (0.44%), and “normal” which encapsulated all remaining sequence (57.44%). The overlap between the three pangenomes was highest in normal (76.8%, 53.6k) and repetitive regions (70.3%, 50.7k), while tandem repeat regions (52.5%, 35.8k) had lower overlap. The most challenging regions for alignment, low mappability (23.0%, 566) and especially centromeric satellites (2.1%, 81), had substantially lower overlap between the pangenomes. This also reaffirms that tandem repeats are disproportionately large contributors to structural variation (Lu et al., 2021).*

**8. Some sentences are kinds of speculations, lacking quantitative measurements. "However, there was a weak increase in edit rate in pggb and cactus pangenomes for HiFi assemblies, potentially related to the increased resolution of complex and repetitive areas beyond centro-/telomeric sequence that cannot be realigned accurately." Please see also "We found that approximately 0.5% and 3.9% of alleles in pggb and cactus were removed during preliminary filtering when only keeping alleles seen in genotypes, suggesting variants may either exist in complex regions that cannot be genotyped confidently or strongly tangled regions that produce spurious variant calls."**

We agree with the reviewer that the first statement was overly speculative for results, and we have removed it. However, we have broadly kept the second statement as we had explicitly quantified the filtering rate and feel results-supported speculation in the discussion based on results is appropriate.

Reviewer #2: This manuscript describes the attempt to create a super-pangenome with bovine species with three pangenome graph construction approaches, pggb, minigraph and cactus. The goal of the manuscript is to evaluate the accuracy of representation of the pangenomes by each approach and make suggestions on different application scenarios.

This manuscript evaluates the three approaches by

1. The computing resources (time and memory) required to construct the data structures

2. The consensus of representation of SVs and SNPs with the assembly-based "truth"

3. General accuracy of base-level representation in edit rate from realigning input assemblies

4. The capabilities of representing polymorphic VNTR loci.

Overall, the manuscript is clear and concise on the metric and objective in each comparison. However, the conclusion drawn from the comparisons are not very convincing for the following reasons:

**(1) for SVs, since there is no ground truth or a validated set of SVs, it is difficult to tell which set of SVs are true SVs.**

We have improved the ground truth with additional optical mapping data, ONT reads, and a simulation analysis (see responses to reviewer 1, point 3).

**(2) A lot of alternative approaches to build pangenomes are overlooked. For example, colored de Bruijn graphs and RLZ-graphs are structures that can also take complete assemblies as input. The output of both genome graph construction schemes can be used with GraphAligner. On the other hand, pangenomes can be represented by not only genome graphs but as a set of strings (see reference flow, Moni and prefix-free parsing works for examples). Without benchmarking with a more diverse set of methods to construct the pangenome, the result of this manuscript is not complete.**

We appreciate the additional tools and approaches suggested, and have added the following text to the introduction to highlight these alternatives in lines 40-42:

*Other pangenome structures that are based on k-mers (Holley & Melsted, 2020), maximal exact matches (Rossi et al., 2022), or other algorithmic compression approaches (Qiu & Kingsford, 2021) are efficient for querying sequences but lack a genomic coordinate system necessary for more general analyses.*

However, tools like Reference flow are not pangenome builders, but rather just an enhanced mapping tool to limit linear reference bias. Our focus is explicitly on constructing pangenomes and specifically graph-like pangenomes based on whole genomes, so other tools like vg are also not appropriate to consider as they focus on augmenting a linear reference with discovered variation. Likewise MONI is an “index” tool designed for MEM-finding in large collections of genomes, rather than building a sequence-based pangenome.

In particular our work focuses heavily on translating between GFA and VCF. The k-mer node nature of ccDBG and rlzgraph (as opposed to nodes representing segments of sequence) means we cannot straightforwardly relate sequence alignment to genomic coordinates. The structure of these graphs reflects this k-mer versus sequence nodes, with much more tangled graphs (Figure R1) and different statistics (Table R1). These structures are also highly efficient at querying presence/absence of specific sequences, but are not suitable for performing coordinate-dependent analyses like eQTL/GWAS/constrained evolution etc.

Figure R1. Left: Bifrost graph (colored compacted De Bruign Graph) covering 750 Kbp (drawn from node 1251 with radius 12). Right: rlzgraph covering 315 Kbp (drawn from node 1251 with radius 10). Drawing either graph with a greater radius caused the BandageNG plot to run out of memory.

Table R1. GFA statistics for chromosome 29, with the number of nodes, edges, and total basepairs. Bifrost is a coloured and compacted De Bruijn Graph.

|  | nodes | edges | bases |
| --- | --- | --- | --- |
| ARS-UCD1.2 |  |  | 51098607 |
| minigraph | 11565 | 16378 | 54361023 |
| PGGB | 4062699 | 5557763 | 58903904 |
| cactus | 4761508 | 6539359 | 58992832 |
| bifrost | 2429250 | 3527058 | 137536488 |
| rlzgraph | 4566307 | 7221766 | 51098607 |

**(3) Given a set of assemblies, there are multiple ways of selecting the samples to be included**

**or held out for analysis. This manuscript only selected one way and does not give a justification for the choice. It would be equally interesting and important to test whether the diversity and the number of the species to be included in the pangenome affect the comparison metrics.**

The assemblies included in the pangenome set were all publicly available, haplotype-resolved bovine assemblies with megabase-scale contiguity along with the reference genome. We later assembled two additional Brown Swiss samples and the parents of the Original Braunvieh sample, but these genomes were assembled with lower coverage and not haplotype-resolved, so we used them for downstream analysis but not for pangenome construction.

We clarified this in lines 84 & 85:

*of which the reference genome and 11 haplotype-resolved assemblies were used for pangenome construction and 8 intermediate-quality assemblies were held out for analysis.*

Some minor comments:

**- It would be very helpful to determine the quality of the comparison results if centromeric and telomeric regions are excluded.**

As addressed in response reviewer #1 point 7, we have better quantified the comparison metrics to account for more fine-grained classification of genomic regions, separating out centromeric satellite regions. However, since many comparison metrics depend on the ARS-UCD1.2 coordinate system (required to surject the pangenome variation into VCF), and the ARS-UCD1.2 reference genome contains limited amounts of centromeric sequence, these regions contributed minimally to the overall metrics. The effect of excluding the centromeric regions entirely from the assemblies is also minor, as observed and discussed through the simulation analysis.

**- In the section "consensus of genomic variation". Are the additionally recovered SVs from the minigraph related to the realignments that are incongruent with graph topology? Seems that minigraph is creating or removing sequences arbitrarily that may result in the additional SVs.**

No, the overwhelming majority of the additionally recovered SVs are consistent with graph topology and originate from true sequence. We have clarified the original text to state this incongruence is negligible on line 127-128:

*Realignment rarely produces paths incongruent with graph topology, where an assembly does not trace through nodes originating from that same assembly, affecting approximately 0.03%, 0.03%, and 0.05% of taurine, indicine, and non-cattle nodes (Supplementary Figure 3).*

**- How does the merging approach influence the overlap of SVs? Is it the different parameters or something else? Please clarify.**

We have clarified the text, removing the ambiguous initial sentence on Line 144:

*The merging approach has an influence on the overlap of SVs between the three pangenomes*

and instead explicitly stated the parameters used in merging

*Merging SVs, allowing for variation in breakpoints up to the minimum of 50% of the SV length and 1 Kbp, identified 221k…*

**- Please add legend to Figure 2(c) or reorder the figures.**

We added an explicit statement to the caption indicating the color scheme for Figure 2c-e are taken from Figure 2b.

**- When they compare optical mapping with the pangenomes, the overlap is low because the OGM is from unrelated samples. Is it possible to calculate the overlap based on the covered breed only? Instead of providing numbers <1%, it would be helpful to see, for the covered breeds, what are the support percentage.**

We thank the reviewer for this suggestion. As addressed to reviewer #1 comment 5, we have reworked this section to reflect the overlap more accurately to only the relevant breed and provide absolute numbers, as the overlapping % can be misinterpreted given the size differences and quantities in SVs called by optical maps and sequencing data.

**- Another important question that comes with using a pangenome is whether using a pangenome improves the short-read/long-read/any-read alignment ratio. It would be good that this work also evaluate this by comparing to aligning to the default linear reference genome.**

Previous work (e.g. <https://www.nature.com/articles/s41467-022-30680-2>, <https://link.springer.com/article/10.1186/s13059-020-02105-0>) has already addressed the advantages of pangenomes over linear references, whereas our current work focuses more on comparing pangenome construction approaches. Aligning to a linear reference versus pangenome will overall have minimal impact, but e.g. missing an alignment to a specific SV not in the linear reference could mean missing an eQTL or GWAS signal, which is much harder to quantify in a single genome-wide metric and beyond the scope of this work. However, we’ve included some additional alignment results using only the linear reference genome as a baseline to highlight the improvement in lines 241-243:

*Aligning to only the linear reference ARS-UCD1.2 resulted in higher edit rates (Bulk: 0.527%, Centro/telo: 3.56%) and lower query coverage compared to pangenomic alignments.*

**- For VNTRs, it would be helpful to give an overview of how each graph stores repeats. Do they identify the repeat motif and create self-loops, or do they store the entire VNTR region as is?**

We thank the reviewer for the interesting suggestion. We had generally observed more frequent looping in pggb and cactus, but we have now thoroughly investigated this for the detailed VNTR example and quantified it more generaly. Minigraph’s definition forbids cycles, leading to VNTR regions appearing similar to genomic regions without repeats. Both pggb and cactus allow cycles, and this is more apparent in VNTR regions where several nodes are repeatedly used. However, due to the complexity of the sequence alignments and forming the graph, as well as potential motif variation, the cycles do not cleanly correspond to multiple traversals of nodes each containing a tandem repeat motif. We have added the following text in Lines 328-333:

*Although the genotyping was similar, bandage plots revealed significantly different graph structure of the VNTR across the three pangenomes (Figure 6c). Minigraph added additional nodes to incorporate the (primarily non-cattle) additional tandem repeats, while pggb, and cactus to a lesser extent, incorporated the additional repeats by looping through the nodes containing the tandem repeat sequence multiple times (****Supplementary Figure 9****). This different representation was observed generally in VNTRs with high repeat counts (****Supplementary Figure 10****).*

As discussed further later, this also highlights the utility of pangenomic analysis of VNTRs. Although we reused an approach to genotype tandem repeat counts through assemblies (still translating the relevant assembly coordinates through the pangenomes), it would be possible to perform such an analysis directly through the pangenomes with currently available tools like odgi.

**- If pangenomes were constructed per-chromosome, would there be missed inter-chromosomal structural variants?**

Yes, constructing pangenomes per-chromosome only captures intra-chromosomal variation and not inter-chromosomal variation. However, we find there is negligible inter-chromosomal variation, as community partitioning (as performed by Gaurracino et al “Recombination between heterologous human acrocentric chromosomes”) clearly groups each chromosome together without suggesting there is meaningful inter-chromosomal homology. Furthermore, pggb (and to a lesser extent cactus) performs all-versus-all alignments, and so building a single genome-wide graph would scale with n^2^ for chromosomes, or approximately 2-3 orders of magnitude slower. We have added some context to the results and discussion to emphasise this in Lines 423-427:

*Building the pangenomes per-chromosome mitigates the quadratic increase in runtime for all-versus-all alignments but neglects inter-chromosomal connections. Building whole genome pangenomes may be necessary when investigating complex chromosomal rearrangements (e.g., Robertsonian translocations) or centromeric homology [16], but such events were not present in the assemblies assessed in this work (****Supplementary Figure 12****).*

Please consider the following questions:

- Are the methods appropriate to the aims of the study, are they well described, and are necessary controls included? If not, please specify what is required.

**The use of a pangenome graph is not essential to many of the analysis (e.g. finding SVs, VNTRs and analyzing regulatory impacts of VNTRs). Especially for VNTR, since the genome graphs are constructed by assemblies, there is no need to conduct a genotyping step as there are ample prior knowledge about the origin of the VNTRs. It is also not necessary to go through the time-consuming steps to construct and align the assemblies in the first place. The entire section about VNTR analyses seems irrelevant to the original challenges identified in the manuscript.**

Pangenomes are effectively just a compact representation of multiple genomes and their alignments, and so there are few analyses that can be done on a pangenome that cannot be recreated on multiple linear genomes. Our analyses focus on validating the accuracy and efficiency of different pangenome building tools through comparisons of SVs, alignment rates, VNTRs, etc., all of which are currently performed analyses on a linear reference genome. However, pangenomic approaches naturally allow identification of similarities and differences directly, rather than performing n^2^ pair-wise analyses. Pangenomes have been established to be useful tools, and it is not our primary focus to demonstrate the power of pangenomes but rather establish strengths and weaknesses of different pangenome builders.

The VNTR analysis is such an example, where we used the pangenomes to directly convert genomic coordinates from one assembly to any other another. While this is possible with n^2^ chain files, we do such an analysis directly through the pangenome GFA files to rapidly identify interesting candidates which we can then analyse further for regulatory impact. With the addition of the 5 genomic region classifications for SV and small variant accuracy, we also provide further analysis on tandem repeats and their accuracy in pangenomes.

We also added text to the discussion to highlight the emerging ability to conduct such analyses directly on pangenomes without involving linear references in lines 479-480:

*Although the eVNTR analysis partially relied on conventional linear alignment approaches, recent advances are enabling purely pangenomic approaches that can simplify and improve analyses [35].*

**Second round of review**

**Reviewer 1:** A point-to-point reply to each question with corresponding line number of text change will facilitate the review process. I managed to find out the authors did enough to satisfy the needs of cattle genomics users. There are some concerns from Reviewer 2's comments. I failed to see authors to fully address them.

**Reviewer 2**

- One advantages of the pan-genome approach, according to the authors in their response, is that the pan-genome approach avoids comparing species by conducting n^2 alignments, which is a valid argument. However, most of the SV validations are still only conducted based on mapping to the reference. It would be more interesting and convincing to see which pangenome graph is better at finding inter-species SVs. For example, how many of these SVs are differences between Yak and Bison assemblies or Taurine and Indicine cattle? And how many of these inter-assembly SVs explains the SVs that are private to each graph?

- Line 196 states that "(pangenomes have) a strong ability to resolve repeat motif variation". In the methods section, it seems that variations are only evaluated based on overlapped regions. How are the motifs evaluated? It seems that comparing repeat motifs require sequence-level comparison. Please clarify.

- There are several grammatical errors. For example, "variation" on line 103 and "sequence" on line 112 should be in plural form. There are other instances of similar typos. Please correct them.

**Authors Response**

**Point-by-point responses to the reviewers’ comments:**

Reviewer reports:

**Reviewer #1: A point-to-point reply to each question with corresponding line number of text change will facilitate the review process. I managed to find out the authors did enough to satisfy the needs of cattle genomics users. There are some concerns from Reviewer 2's comments. I failed to see authors to fully address them.**

Reviewer #2:

**- One advantages of the pan-genome approach, according to the authors in their response, is that the pan-genome approach avoids comparing species by conducting n^2 alignments, which is a valid argument. However, most of the SV validations are still only conducted based on mapping to the reference. It would be more interesting and convincing to see which pangenome graph is better at finding inter-species SVs. For example, how many of these SVs are differences between Yak and Bison assemblies or Taurine and Indicine cattle? And how many of these inter-assembly SVs explains the SVs that are private to each graph?**

This is a complex question. The tool we use to merge SVs, Jasmine, merges on a per-variant level, and so does not keep track of which genotypes from different VCFs intersect (and the - -output_genotypes option of Jasmine does something different). So determining whether an SV is inter-assembly (per-graph) or mutual to different graphs (across any assembly) is mutually exclusive. Conducting a more fine-grained analysis would potentially require analysing pangenome-intersections over 2047 separate VCF subsets (each of the 2^11 combinations of inter-assembly genotype overlaps with at least one non-reference genotype).

However, this type of question was previously addressed for a smaller pangenome (see Figure 2A in<https://www.pnas.org/doi/10.1073/pnas.2101056118>), and we already show similar results in this manuscript in Figure 2b and Figure 3d, showing the per-assembly breakdown of SVs and small variation is fundamentally highly similar between the pangenome graphs for each of the assemblies across different groupings (taurine cattle, indicine cattle, and non-cattle). There are some differences, particularly for SVs between cactus and the other pangenome tools, but this is already a point broadly reflected many times throughout the paper. We also show in Figure R1 below that each graph has highly similar inter-assembly intersections (up to allele-frequency arguments for minigraph discussed already in Figure 2C as minigraph does not create new alleles for SVs with moderate/high sequence similarity). Combined with the already presented results for comparable SV F-scores, we believe these results already demonstrate no tool has a particular advantage or bias for finding inter-(sub)species SVs.

*Figure R1. SV intersections across assemblies for 65 of the most frequent intersections out of 2048 possible.*

**- Line 196 states that "(pangenomes have) a strong ability to resolve repeat motif variation". In the methods section, it seems that variations are only evaluated based on overlapped regions. How are the motifs evaluated? It seems that comparing repeat motifs require sequence-level comparison. Please clarify.**

This sentence refers to the fact that pggb and cactus have F-scores of 0.93 and 0.89 respectively for small variation in regions annotated as tandem repeats, which is only slightly weaker than in “easy” regions. As such, these pangenomes (given they can accurately represent SNPs) *can* resolve different motifs. Being unable to accurately represent SNPs in tandem repeat regions means that differences in motifs could easily be explained by errors, which we have already established is not the case.

We have added some additional context to clarify that having accurate SNP-level representation is important for distinguishing motifs, which may only differ by a SNP:

...indicating a strong ability to resolve repeat motif variation which may only differ by a single or a few bases.

We believe our intent is correctly represented by this text (that it is possible), and should not be interpreted as an explicit analysis that resolves repeat motifs.

**- There are several grammatical errors. For example, "variation" on line 103 and "sequence" on line 112 should be in plural form. There are other instances of similar typos. Please correct them.**

We believe several of these are correct, as variation and sequence in this context are effectively uncountable nouns. Where we do refer to either term as countable, we already use the plural forms (e.g., autosomal sequences [since there are 29]). However, we will accept the style as recommended during copy editing.
